# Supplementary material for: Optical Control of the Localized Surface Plasmon Resonance in a Heterotype and Hollow Gold Nanosheet
Source: Nanomaterials (Basel). 2023 Jun 8;13(12):1826. doi: 10.3390/nano13121826 (PMC10301572; doi:10.3390/nano13121826)
Supplement: Supplementary file 1 [file nanomaterials-13-01826-s001.zip › nanomaterials-2422324-supplementary.pdf]

Supporting Information

Supplementary FDTD calculations which could support some of our conclusions mentioned in the main text.

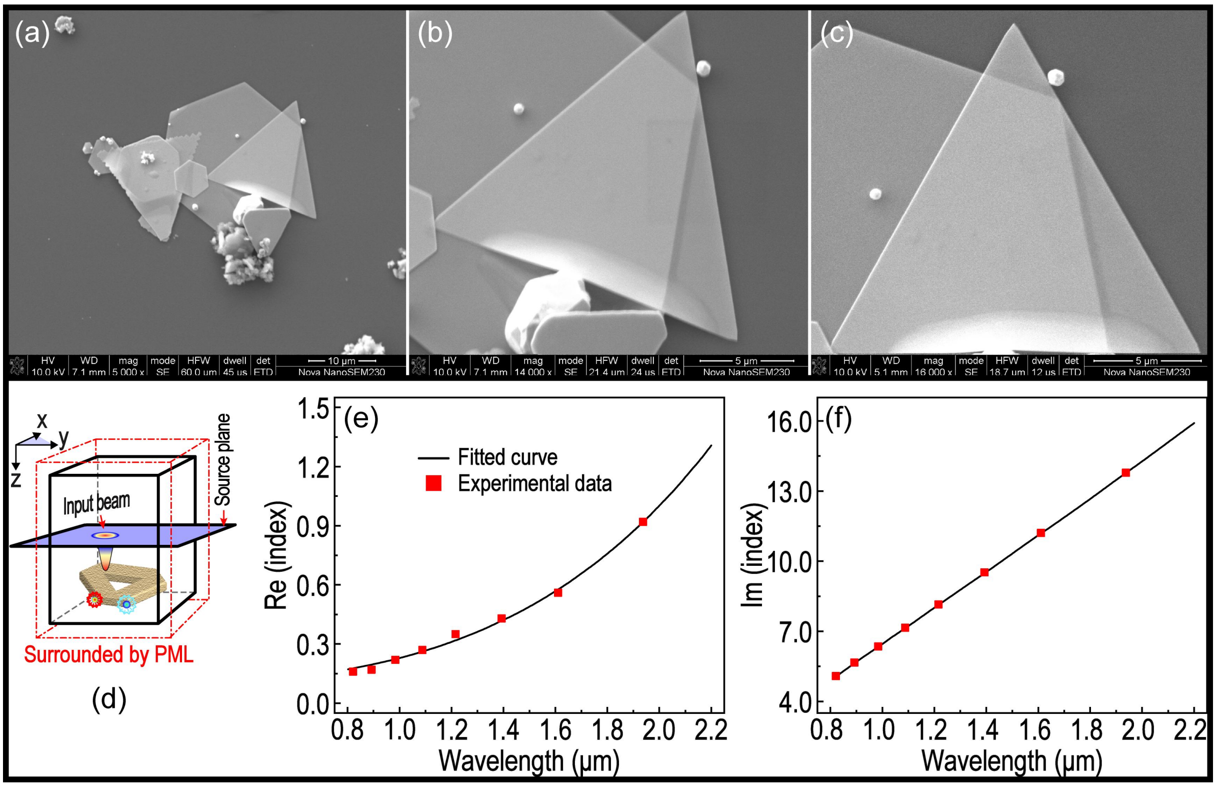

Figure S1. SEM images of solid gold nanosheets (SGNSs) and Schematic illustration of the FDTD computational domain and the fitted materials' data. (a)-(c) SGNSs at different magnifications. (d) FDTD computational domain; (e) and (f) A comparison of the fitted curve and the experimental data for FDTD simulations.

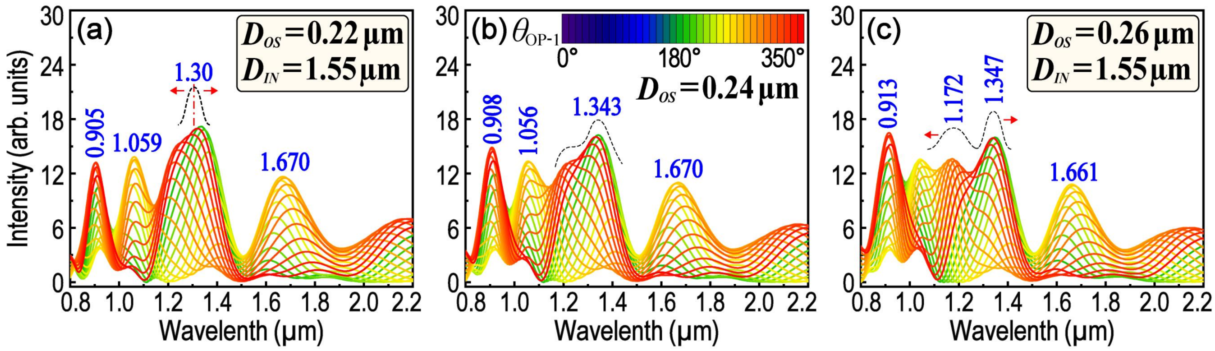

Figure S2. A comparison of the polarization-dependent LSPR spectra. (a) $D_{OS} = 0.22\mu m$; (b) $D_{OS} = 0.24\mu m$; (c) $D_{OS} = 0.26\mu m$; The peak-splitting (~ 0.3 μm) could be observed clearly when D_{OS} varies from 0.22 μm to 0.26 μm.

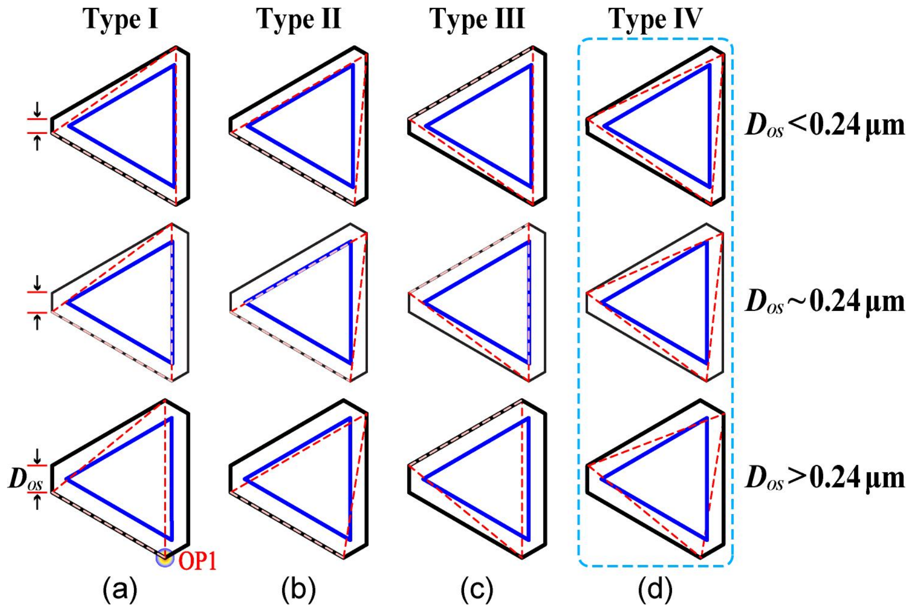

Figure S3. Four types of T-T HGNSs resolved from H-T HGNS as respect to OP1. A red dashed-line triangle cooperates with a blue solid-line triangle which constructs a T-T HGNSs. (a) Type I; (b) Type II; (c) Type III; (d) Type IV;

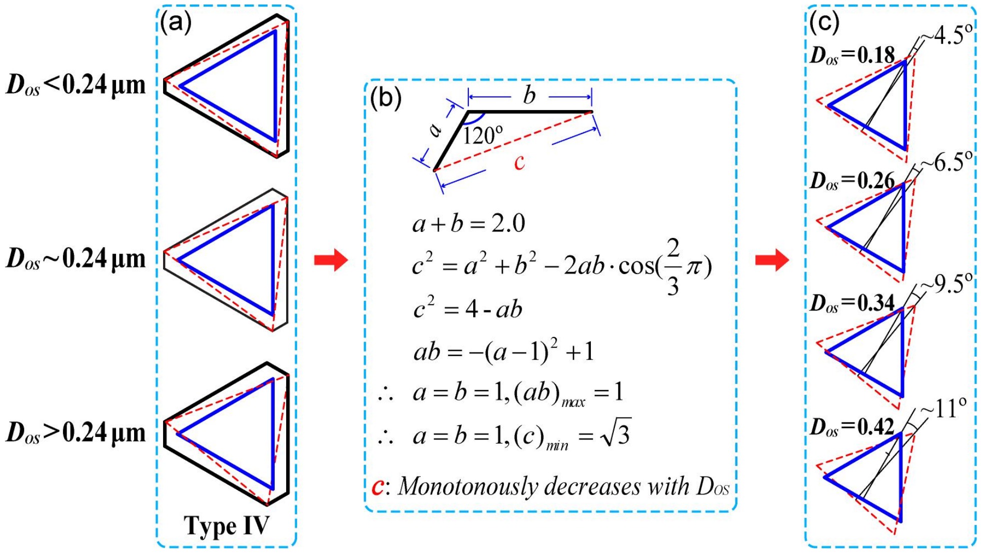

Figure S4. Illustration of the variation tendency of T-T HGNS (Type IV) with the increase of D_{OS}. (a) Sketch map of T-T HGNS (Type IV); (b) Calculation of the side length of red dashed triangle. The length decreases monotonously with the increase of D_{OS}; (c) The difference of the spatial orientation between two cooperated triangles. The included angle increases monotonously with the increase of D_{OS}.

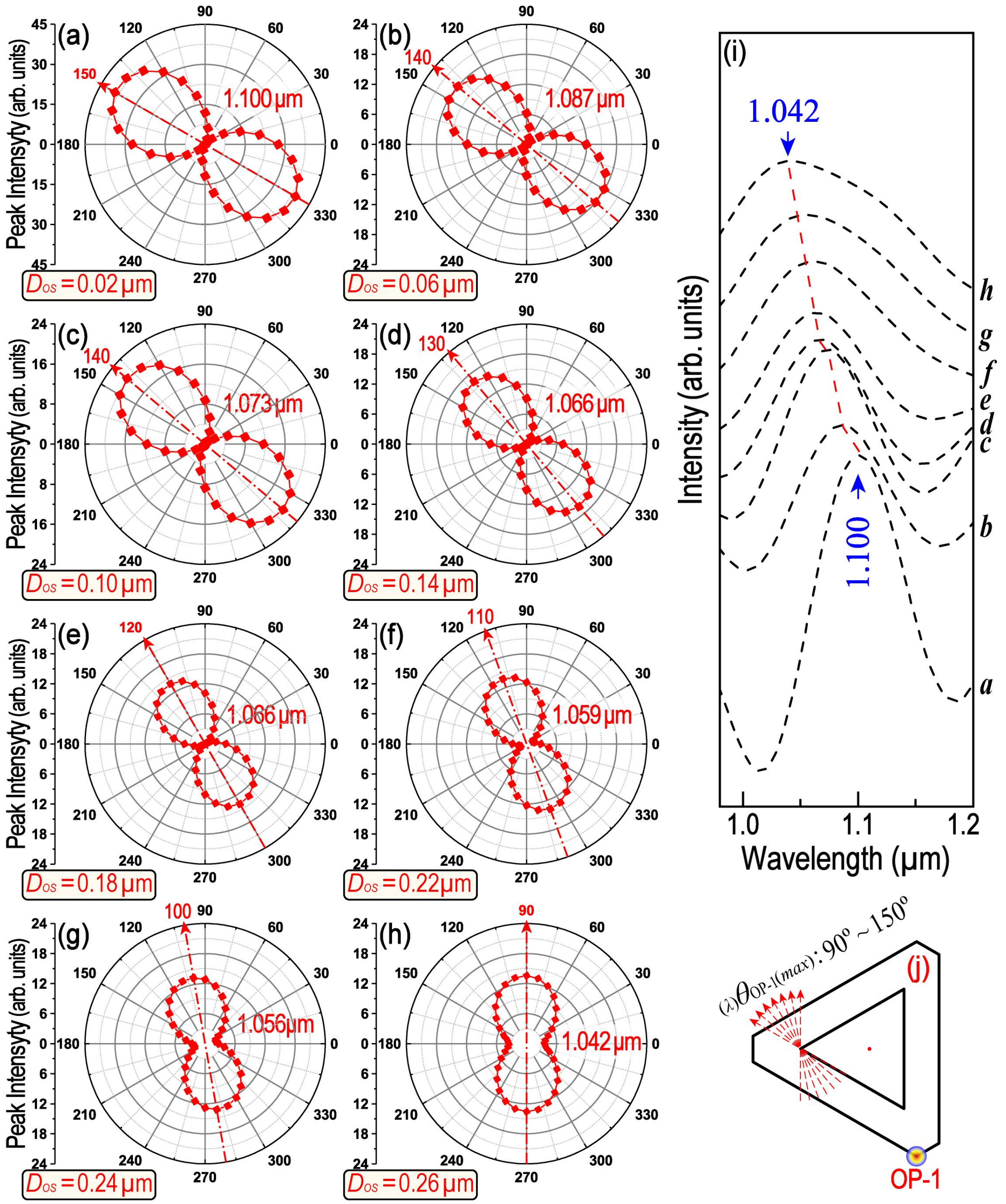

Figure S5. Polarization characteristics of LSPR peak around 1.042-1.100 µm. (a)-(h) Rotation trend of $_{(\lambda)}\theta_{OP\text{-}1(max)}$ with the increase of D_{OS} (from 0.02 to 0.26 µm); (i) Shift of the LSPR peak wavelength at their respective $_{(\lambda)}\theta_{OP\text{-}1(max)}$. Italic small letters *a*-*h* correspond to (a)-(h); (j) $_{(\lambda)}\theta_{OP\text{-}1(max)}$ varies within 90º - 150º.

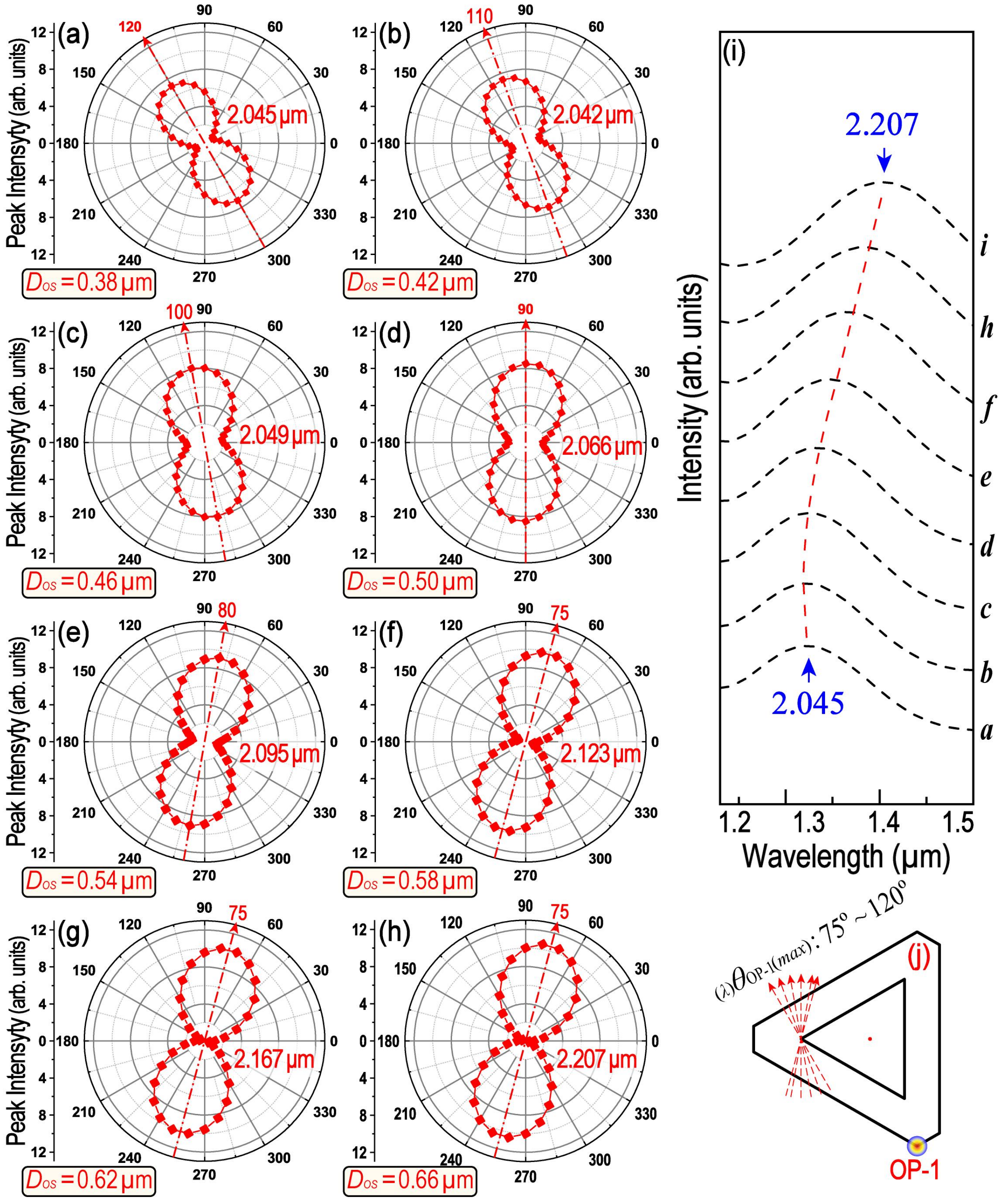

Figure S6. Polarization characteristics for LSPR peak around 2.045-2.207 μm. (a)-(h) Polar plots as a function of θ. D_{OS} increases from 0.38 to 0.66 μm. (i) Shift of LSPR peak at their respective $_{(\lambda)}\theta_{\text{OP-1}(max)}$. Italic small letters *a*-*i* correspond to (a)-(i); (j) $_{(\lambda)}\theta_{\text{OP-1}(max)}$ varies within 75° - 120°.

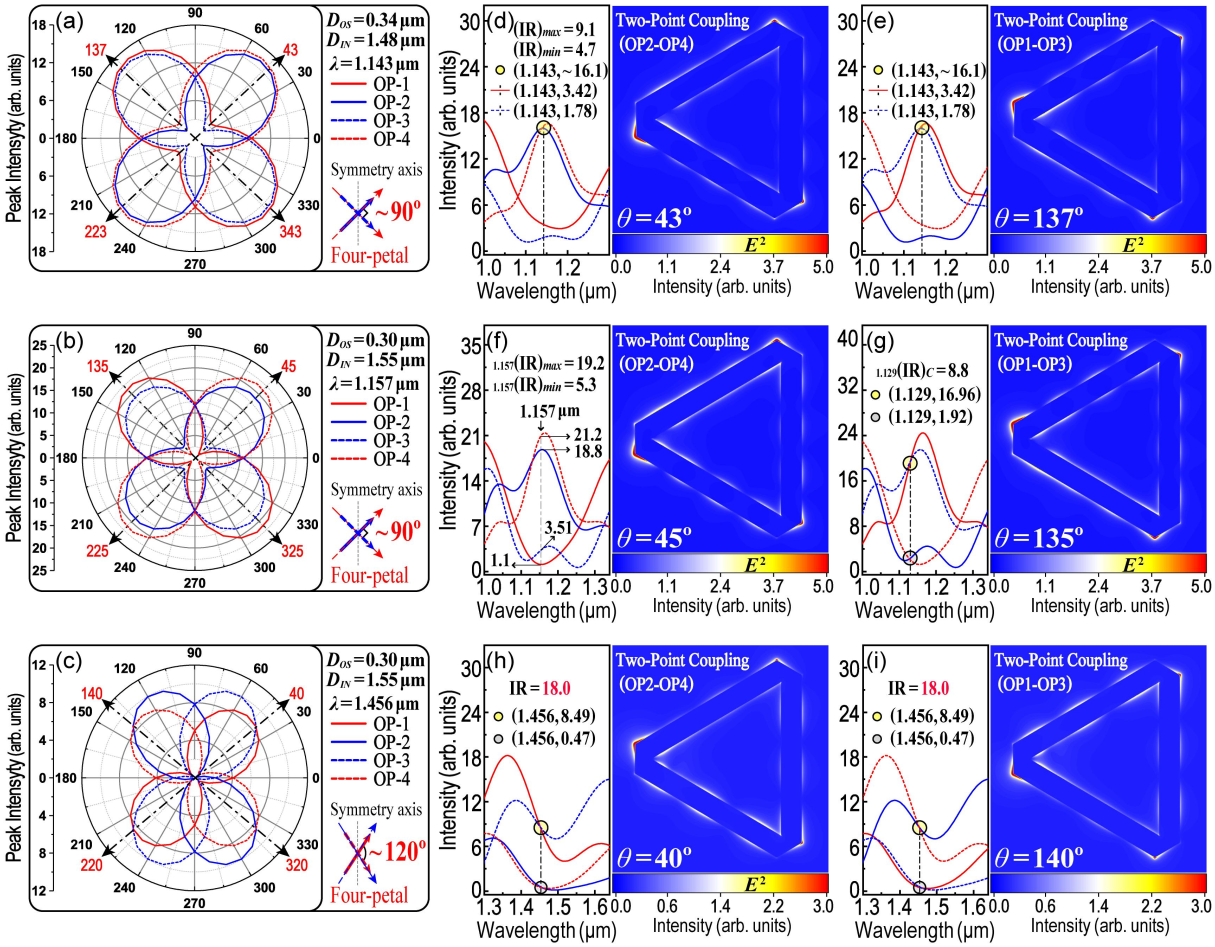

Figure S7. A comparison of the two-point coupling of LSPR. (a)-(c) Four-petal or quasi Four-petal polar plots at different groups of parameters; (d)-(i) Two-point coupling at different parameters.

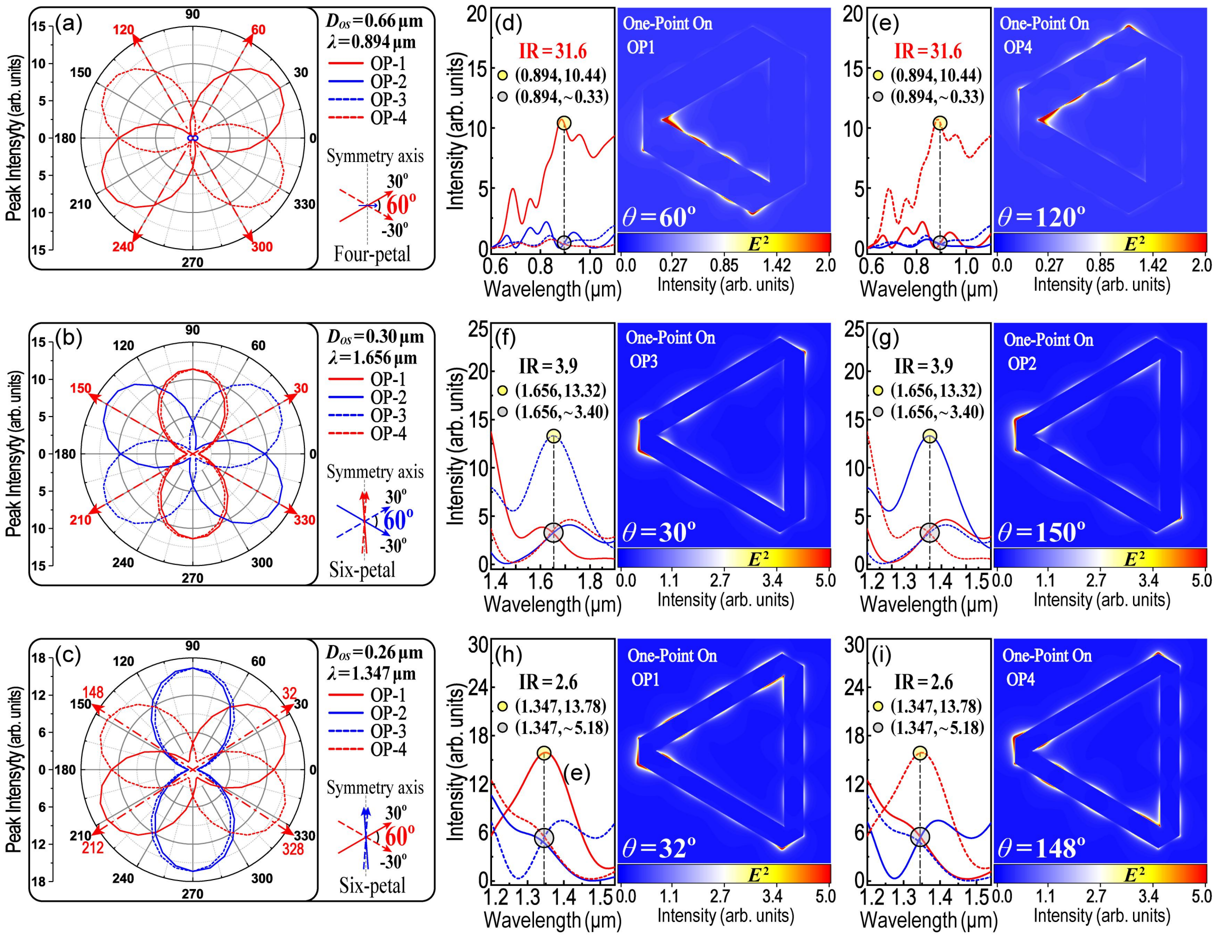

Figure S8. One-point on/off switching of LSPR. (a) A special Four-petal polar plot; (b),(c) Six-petal polar plot as Figure 8(a) and Figure 9(a) in the main text; (d)-(i) One-point on using different parameters and their field map.
